# Contradistinctive floral attributes, pollination guilds and their consequence on the outcrossing rate in two elevational morphs of *Rhododendron arboreum* Sm.

**DOI:** 10.3389/fpls.2024.1355680

**Published:** 2024-03-28

**Authors:** Sachin Singh Sorokhaibam, Atika Chandra, Ratul Baishya, Saroj Kanta Barik, Shailendra Goel, Rajesh Tandon

**Affiliations:** ^1^ Department of Botany, University of Delhi, New Delhi, India; ^2^ Department of Botany, Maitreyi College, University of Delhi, New Delhi, India; ^3^ Department of Botany, Centre for Advanced Studies in Botany, North-Eastern Hill University, Shillong, India

**Keywords:** floral trait, floral evolution, mating system evolution, pollination ecology, reproductive biology, SSR, tree

## Abstract

Infraspecific floral trait variations may appear in response to elevational differences in alpine plant species. There is enormous information on the selection of such morphs mediated by biotic and/or abiotic variables. Whether such differences contribute to differences in reproductive strategy and mating outcomes is rarely investigated. We investigated these aspects in two distinct elevational floral morphs (Red and Pink) of *Rhododendron arboreum* Sm. in Western Himalaya. The red morphs occupy the lower elevations while pink morphs the higher elevations. The two morphs differ in floral traits like phenology, dimension, display, quality of floral rewards, and pollinators that happen to influence interaction with available pollinator pool at each elevation. The pink morph exhibits entomophily, while the red ones show ornithophily. Although experimental pollinations established that both the morphs are self-compatible, selfing results in significantly lower fruit-set than either cross- or open-pollinations. The outcrossing rate in the red morph, as determined by using simple sequence repeat (SSR) markers, was higher (t_m_=0.82) than that in the pink morph (t_m_=0.76), with a tendency of the latter to be shifting towards mixed-mating strategy. However, the extent of biparental inbreeding was comparable among the two morphs. It is inferred that the differences in the mating outcomes among the morphs in the tree species are linked to those emerging from floral traits and the pollination by different functional groups of floral visitors.

## Introduction

Knowledge of elevational differences in floral traits and its consequent influence on the mating system is crucial for understanding plasticity in the reproductive strategy of alpine plant species (Fabbro and Körner, 2004). In animal-pollinated plants, floral contrivances such as architecture, display, olfaction, and rewards can vary significantly across relatively short vertical distances along the elevational gradient (Basnett et al., 2019). Such variations may emerge in response to variations in temperature and the available pool of pollinators (Lefebvre et al., 2018). Additionally, floral traits also shape interactions with effective pollinators and gene-flow patterns (Junker et al., 2013). Studies conducted in multiple mountain ranges have provided evidence regarding floral polymorphism and associated pollinators across elevational gradients, both at the community and species levels (Adedoja et al., 2018; Maguiña-Conde et al., 2023). However, a limited number of studies have investigated how floral traits, pollinator pools, and mating outcomes may vary with elevation within a species, especially in the Himalayan region (Gurung et al., 2018).

Infraspecific variation in floral traits (shape, size, color and floral reward) and their display (number and arrangement of flowers) in a geographic mosaic reflects adaptation of different populations to diverse pollination conditions (Miller, 1981; Newman et al., 2012). While it is evident that abiotic factors, herbivores, and other neutral processes can contribute to the emergence of differences in floral traits, the primary explanation for floral variation is often attributed to geographic patterns or the available composition of pollinators (Grant and Grant, 1965; Stebbins, 1970). Variations in floral traits may also determine the visitor’s spectrum and effective pollinators (Basnett et al., 2019; Layek et al., 2022). Some earlier studies have indicated that by engaging local pollinators at different elevations, variations may emerge in floral traits of the interacting plant species. For example, in *Costus guanaiensis* var. *tarmicus* (Loes.) Maas (Costaceae), differences in floral traits and pollinator assemblages indicated a transition to pollination by hummingbirds from bees (Maguiña-Conde et al., 2023). In *Trollius ranunculoides* Hemsl. (Ranunculaceae), the study revealed a significant decline in bee visitation rates with increasing altitude (Zhao and Wang, 2015). A closely similar study on *Campanula punctata* var. *hondoensis* (Kitam.) Ohwi ex T. Shimizu (Campanulaceae) and *Prunella vulgaris* L. (Lamiaceae) showed a noticeable geographic variation in pollinator assemblages, especially changes in pollinator size along an elevational gradient accompanied by altitudinal floral size changes (Nagano et al., 2014; Kuriya et al., 2015). The occurrence of a geographic mosaic of locally adapted floral morphs in these species, even at a finer spatial scale, indicates that floral size is subject to selection mediated by a diverse pool of available pollinators (Kuriya et al., 2015).

Challenging environments also have a significant impact on plant reproduction through various mechanisms apart from selection on floral traits (Escaravage et al., 1997). In alpine regions, unfavorable environmental conditions often lead to reduced diversity and abundance of pollinators. Such demographic shifts may impact reproductive efficiency of plants (Kudo, 1993). In regions characterized by a limited presence of pollinators, plant reproductive success is significantly hampered, primarily because of pollen/pollinator limitation (Larson and Barrett, 2000). However, many studies have indicated that at higher elevation, plants may undergo alternative pollination strategies, with self-pollination emerging as a crucial method for reproductive assurance (Abdusalam et al., 2022). This adaptation is driven by, limited and unpredictable pollinator services, and the short growing seasons (Körner and Paulsen, 2009).

*Rhododendron* (Ericaceae) is one of the speciose genera (~1000 species) with distribution across the northern temperate zone, tropical southeastern Asia, and northeastern Australia (Chamberlain et al., 1996). Many *Rhododendron* species are found across a broad range of altitudes as well. These species exhibit significant diversity in their flowering patterns and floral traits, which attracts a range of generalist pollinators including bees, flies, butterflies, and birds (Kudo et al., 2011; Huang et al., 2017). Given their extensive distribution and notable variation in the floral traits, rhododendrons represent an ideal model system for examining how floral attributes and interactions between plants and pollinators can have different outcomes on their net reproductive outcomes in response to shifts in elevations.

*Rhododendron arboreum* Sm., a Himalayan tree species, shows a shift in flowering phenology in response to differences in mean air temperature along an elevational cline (Gaira et al., 2014; Lamsal and Welch, 2016; Tandon et al., 2020). The current trend of rise in winter-spring temperature and the observable early flowering suggests that *R. arboreum* might widen its distributional range in response to local shifts in climatic patterns due to global warming (Ranjitkar et al., 2013). Moreover, in *R. arboreum*, there is a noticeable difference in floral color at different elevations. At lower altitudes, the trees exclusively bear red flowers, and those at higher altitudes, the trees bear pink flowers (Ollerton et al., 2020). However, whether these color morphs differ in other reproductive traits as well, has not been investigated so far. Especially there is a paucity of information, whether differences in the floral traits among morphs can lead to variation in pollinators and mating pattern at the different elevations. Mating system essentially establishes the proportion of self and outcross progeny in a fruit or a genet, which can only be ascertained by employing molecular markers (*see* Barman et al., 2018).

We looked into the reproductive biology of the two distinct floral morphs of *R. arboreum* occupying different elevational niches in the Western Himalayas. More specifically, we recorded differences in floral phenology, floral biology, web of plant-pollinator interactions, and breeding system of the species. Importantly, we also looked into the differences in mating outcomes of the morphs by employing SSR markers in the species for the first time. These findings have broader implications in building our understanding of evolution between plants and pollinators, as well as conservation of the alpine tree species.

## Materials and methods

### Study-sites and morphs

The study was carried out on the trees located at two different elevations in the protected forest area of Kedarnath Wildlife Sanctuary (KWLS), Uttarakhand, India during the peak flowering-time in the years 2021 and 2022. We selected the two elevations as representative populations of the two color morphs of the tree namely, Triyuginarayan (Red Morph), (30°38′5′′N, 78°58′43′′E, 2000 m AMSL) and Chopta (Pink Morph), (30°29′10′′N, 79°12′5′′E, 3000 m AMSL).

### Floral phenology

Flowering phenology was recorded at the population level at both the elevations, and it was designated at peak when >50% of the trees were in bloom. At the individual tree level, ten flowering twigs were randomly selected on each of the marked trees (n=10, each elevation) and regularly monitored, every alternate day during the flowering period and subsequently at 10-days interval till seed dispersal, for various phenoevents namely onset of flowering, peak flowering period, fruiting period, and dispersal of seeds. For recording the onset of anthesis, flower buds (n=20) of similar stages were marked around 07:00 h and subsequently observed at intervals of every 3 h until their opening.

### Floral biology

Different stages of the flower were recognized (**Supplementary Table 1**) and monitored for recording the floral mechanism like timing of anthesis, herkogamy and dichogamy. The onset of receptivity of the stigma was ascertained by localizing peroxidase activity (Galen & Plowright, 1987), on the different stages of flowers. The activity of the enzyme was determined in terms of number of oxygen bubbles released per minute. The viability of fresh pollen grains was estimated by FCR test and fertility with the help of acetocarmine (1%) (Shivanna and Rangaswamy, 1992).

The outcrossing-index (OCI) was determined by recording the flower diameter, presence of herkogamy and/or dichogamy (Cruden, 1977). For this purpose, flowers (n=20) were randomly selected from each population. Dimensions of corolla, stamen and pistil were measured using a digital vernier-calliper and a plastic ruler. From these morphometric details, the existence of herkogamy was examined. Incidence of dichogamy was ascertained by recording the duration of stigma receptivity vis-a-vis onset of anther dehiscence and pollen viability data from different floral stages. For computing the pollen:ovule ratio (a measure of the possible type of breeding system), another set of randomly selected flowers (n=20, each population) was used. Pollen and ovule production in a flower was determined by following the method described by Dafni et al. (2005).

### Breeding system

The breeding system of the species was determined through experimental pollinations (Shivanna and Tandon, 2014). Based on the data gathered on pollen viability and the stigma receptivity, we performed four pollination treatments: (i) Apomixis (n=150, each population): bagging the unpollinated and emasculated flowers, (ii) Spontaneous-Autogamy (n=150, each population): bagging the flowers without emasculation and facilitated pollination, (iii) Facilitated-Autogamy (n=220, each population): pollination among flowers of the same tree without emasculation and, (iv) Xenogamy (n=220, each population): pollination among flowers from different trees after emasculation of the receptive flower. Additionally, some random, undisturbed flowers were tagged and observed for natural fruit-set through open-pollination (n=250 per population). For pollination, flower stages (R3 and R4) were employed (**Supplementary Table 1**), and for bagging, butter-paper bags (22.5cm x 16.5cm) were used throughout the study. The resultant values were expressed in terms of percentage fruit-set.

The index of self-incompatibility (ISI) was calculated as the percentage of fruits obtained from self-pollination treatment divided by those obtained through xenogamy (Zapata and Arroyo, 1978). A species is considered fully self-incompatible when the ISI value is <0.2, partially self-compatible when it is >0.2 but <1, and considered self-compatible, when it is ≥1. The reproductive efficacy index was computed as the percent fruit-set from open pollination divided by that from xenogamy (Zapata and Arroyo, 1978). The pollen limitation index (L) was expressed as L = 1 - (P_o_/P_s_), where P_o_ is the fruit-set (%) through open-pollination and P_s_ represents the fruit-set (%) through xenogamy (Larson and Barrett, 2000). Inbreeding depression was estimated as ID = 1 – (ω_s_/ω_o_), where ω_s_ and ω_o_ refer to the fitness (percentage fruit-set) of the fruits formed by autogamy and xenogamy, respectively (Charlesworth and Charlesworth, 1987).

### Floral visitors

The identity of floral visitors and observation of their foraging activities were recorded during the peak-flowering time in both the seasons of the study period. The observations were confined to only those visitors that foraged the flowers for reward. In order to ascertain the foraging behavior of floral visitors, we made direct observations on marked trees (n=10) in full-bloom from each population and recorded their interactions in terms of visitation time (time of the day), flower-handling time, number of flowers of a tree visited and the number of trees visited in a single foraging bout by each type of floral visitor. For counting the number of trees covered in a bout, forager of each type was monitored from the time of initiating foraging on any of the marked tree and then tracking it, with the aid of binoculars, until it settled for more than 20 sec without foraging. We recorded the legitimate foraging visits (visits leading to contact with the stigma) and expressed it as an indirect measure of efficacy of the visitors to deposit pollen. The observation period was staggered over a span of one-week at each site, and observations were recorded in batches of 5 h per day, spanned across regular intervals of 60 min, for 60 min duration between 06:00h and 18:00 h. The observation batches were altered to cover the entire duration of diurnal foraging activity. The floral visitors were photographed using a digital camera (Nikon Coolpix A900).

### Nectar analysis

The average standing-nectar volume was quantified using single-channel micropipette (Eppendorf, 10-100 µl). Total sugars were quantified using a hand-held portable refractometer (Sugar/Brix Refractometer 0 to 80%, Sper Scientific). For estimation of amino acid content, the nectar sample was loaded onto the Whatman filter paper (No.1), dried and sprayed with 0.2% ninhydrin solution. The filter paper was kept at 65°C for 30 min. The color intensities of the nectar spots were compared with the histidine calibration scale (Dafni et al., 2005). The presence of alkaloids, phenolics and proteins in the nectar was analyzed qualitatively by using the colorimetric method. For this, Whatman (No. 1) filter paper strips were loaded with nectar samples, air dried and tested for the presence of the different components. The development of yellow, orange, or red color after adding Dragendorff’s reagent to the dried nectar spot indicated the presence of alkaloids. For the detection of phenolics, 0.5N of Folin Ciocalteu’s Reagent was added, dried again and a drop of 20% Na_2_CO_3_ was added; development of a brown color indicated the presence. For proteins, 0.1% bromophenol blue was added, dried, and washed in three changes of 5% acetic acid. Presence of proteins was indicated by the development of bluish-green color (Dafni et al., 2005).

### Outcrossing rate

For determining the outcrossing rate (OCR), six mother plants were randomly selected from each site, ensuring that they were located at a distance of at least 50 m from each other. Leaves were sampled from each of the mother plants in liquid nitrogen. Also, from each of the mother plants, mature but un-dehisced fruits (capsule, n=20) were collected in different paper pouches. These capsules were made to dehisce in the lab under the heat of a table lamp (35-40°C) for 24 h. Once dehisced, the seeds were carefully transferred to a 2 ml microcentrifuge tube and kept at -20° C for 20-30 days before germination.

#### Seed germination

Before germination, seeds from all the sampled capsules from each mother tree were pooled. The seeds were germinated on moistened peat-moss laid on plastic trays. The trays were covered with thin, transparent plastic sheets that were sporadically perforated with a sharp needle. The trays were kept in partial sunlight. It took nearly 20-30 days for the germination to occur. After nearly 45 days of germination, the seedlings were transferred to paper cups containing peat-moss to avoid crowding.

#### Extraction of DNA

Nearly 10 mg of leaf from each individual (~6 months after germination) was used for DNA extraction. The genomic DNA was extracted using a mini-prep DNA isolation method (modified from Murray and Thompson, 1980). The whole seedling was crushed in a 1.5 ml microcentrifuge tube containing 100µl of CTAB extraction buffer, by using a plastic micro-pestle. After crushing, 400µl of buffer was added along with 5µl of RNase and incubated at 65°C for 1 h. Then, 500µl of Chloroform: isoamyl-alcohol (24:1) was added, mixed well for 10 min., and subjected to centrifuge at 14,000 rpm for 2 min. The supernatant was collected in a fresh tube. Subsequently, 1/10 volume of sodium acetate and 2 volumes of ethanol were added and kept at -20°C for 20-30 min. The tubes were then centrifuged at 14,000 rpm for 5 min at 4°C. Subsequently, the supernatant was discarded and the DNA pellet was washed in 70% ethanol. The pellet was then air-dried and dissolved in nuclease-free water (Sigma water). The extracted DNA was stored at -20°C until further use.

DNA from the leaves of the mother plants were extracted using a modified CTAB method (Murray and Thompson, 1980), with minor modifications (100 mM Tris HCl, pH 8.0; 20 mM EDTA; pH 8.0; 2% CTAB; 1.4 M NaCl; 1.5% β-mercaptoethanol; 1% PVP). The purified genomic DNA was dissolved in nuclease-free water and stored at -20°C until further use.

#### SSR markers and PCR reaction

Ten progenies per mother plant were used for the PCR. The information on SSR primer pairs used for analyzing the mating system was obtained from (Sharma et al. (2020); **Supplementary Table 2**), and were synthesized by Eurofins Genomics, India. PCR amplification was carried out in a total volume of 15 µL reaction mixture consisting of 1X PCR buffer (10 mM Tris-HCl, 50 mM KCl, 0.1% Triton X-100), 1.5 mM MgCl_2_, 160 µM of each dNTPs, 0.2 µM each of forward and reverse primer pair, 0.2 U Taq DNA-polymerase (Edna biolabs) and 35-40 ng of template DNA. The PCR routine for DNA polymerization comprises one denaturation cycle at 94°C for 4 min, followed by 35 cycles of 94°C for 40 sec, annealing at optimum temperature (Ta, which are different for each primer pair) for 30 s and extension at 72°C for 40 s. The final extension step was carried out at 72°C for 8 min. The amplified PCR products were separated on a 6% polyacrylamide gel in 1X TBE buffer by using a vertical electrophoresis unit (Benchtop Lab Systems). The bands on the gel were visualized by using the Gel-Documentation System (Vilber Bio-Print). DNA ladder mix (GeneDireX) was used for analyzing DNA fragment size.

### Data analysis

Normality of the data was verified using the Shapiro-Wilk test. Data in percentage were square-root arcsine transformed for achieving homoscedasticity. To check whether the differences in the mean values of different attributes of the two populations were significant, Student’s t-test was performed. We applied a three-way ANOVA test (General Linear Model) to ascertain the effect of pollination treatments, morphs and seasons (independent variables) on the fruit-set (dependent variable). One-way ANOVA was performed to compare the differences in the fruit-set resulting from different treatments. For estimating the OCR, the data gathered after scoring the bands was analyzed using MLTR program version 3.4 (Ritland, 2002), and based on a multi-locus mixed-mating model (Ritland and Jain, 1981).

## Results

### Flowering phenology

The two floral morphs of *R. arboreum* exhibited variation in the onset of flowering time. At lower elevations (red morphs), flowering was initiated by fourth-week of January and attained peak by February fourth-week, while at higher elevations (pink morphs), flowering was initiated by the end of February and it peaked (>50% of the canopy in bloom) by the fourth-week of March/first-week of April. The flowering culminated among the red morphs by the middle of April, while in pink morphs, it extended till the first-week of May. The duration of fruiting period did not vary among the two morphs; they were initiated by the fourth-week of May, and began to disperse seeds by the fourth-week of September. In both the morphs, the time taken for anthesis was more than 2 days from bud break - 55.5 ± 0.7 h (51-60 h) in red morphs and 59.1 ± 0.7 h (54-63 h) among the pink morphs.

### Floral biology

*Rhododendron arboreum* is characterized by the presence of terminal racemes in a bunch. The two morphs distinctly varied in floral features while the extent of pollen viability and fertility did not show significant differences (**Supplementary Table 3**). There were 10 stamens in each flower with one longest, one shortest and four pairs of intermediate lengths. In both the morphs, although the flowers became receptive 24 h before anthesis, the anthers dehisced only after the complete opening of flowers. Also, the anthers exhibited poricidal dehiscence and pollen grains released as tetrads, intertwined with viscin threads. However, the nectar guides were more distinct in the pink flowers.

#### Outcrossing-Index

The mean number of pollen grains in a flower in red and pink morphs were 858900 ± 11976 and 655750 ± 8459, respectively, while that of ovules was 3670 ± 39 in red and 2866 ± 55 in pink morph. Pollen:ovule ratio in the red morphs was 234.7 ± 5 and that in pink was 230.6 ± 6; the range suggests that both morphs exhibited facultative-xenogamy. On the other hand, the OCI value, obtained by summing up the corolla width (3), dichogamy absent (0) and presence of herkogamy (1) for both the floral morphs turned out to be 4, which indicated the prevalence of xenogamy or outcrossing in the tree species.

### Breeding system

Experimental pollinations established that *R. arboreum* is a self-compatible species (**Table 1**). Flowers bagged to ascertain agamospermy and spontaneous autogamy failed to set fruits in both morphs. There were negligible differences in fruit-set resulting from the same treatment in the same morph between different seasons (F*_(2,126)_* = 0.047; p = 0.954; three-way ANOVA). The amount of fruit-set resulting from facilitated self-pollination (~53% in red and ~57% in pink morph) was significantly lower (F*_(2,135)_* = 54.115, P < 0.001; one-way ANOVA) than that from facilitated crossing (~85% and ~78% in red and pink morphs, respectively) as well as open-pollination (~74% in Red and 70.84% in Pink morph) in both the seasons. Moreover, fruit-set from facilitated cross-pollinations was greater than that from open-pollination in both the populations. Fruit-set resulting from open-pollination or xenogamy, was more in the case of the red morphs than the pink ones. The indices of self-incompatibility (ISI) and reproductive efficacy were more in the pink morphs while inbreeding depression (ID) was higher among the red morphs (**Supplementary Table 4**).

**Table 1:** Results of fruit-set (%) recorded from different pollination treatments in *R. arboreum*.

Treatment	2021		2022	
	Red Morph	Pink Morph	Red Morph	Pink Morph
Apomixis	0 (100)	0 (100)	0 (50)	0 (50)
Spontaneous-Autogamy	0 (100)	0 (100)	0 (50)	0 (50)
Facilitated-Autogamy	54.17 (120)	58.33 (120)	53.00 (100)	57.00 (100)
Xenogamy	85.83 (120)	77.50 (120)	84.00 (100)	79.00 (100)
Open-pollination	75.00 (100)	71.00 (100)	73.33 (150)	70.67 (150)

The numbers inside the parentheses indicate the sample sizes.

### Floral visitors

**Figure 1:**
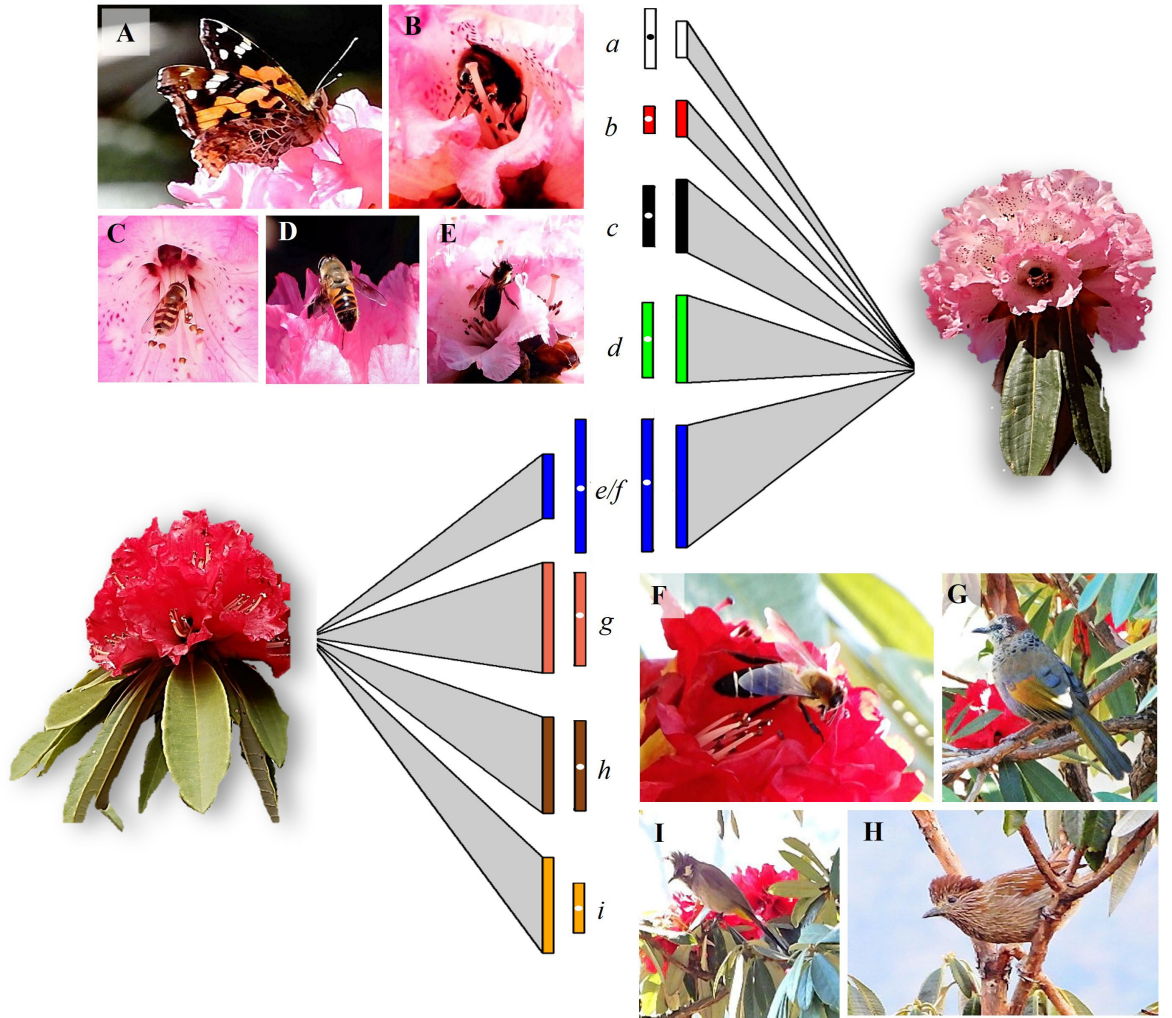
Details of pollinators and their foraging behavior (visits/tree and flower-handling time) associated with the two elevational morphs of *Rhododendron arboreum*. Left and right panels show the different pollinators of the pink and red morphs **(A–I)**, respectively. The size of the triangle (link) connecting the morph represents the relative strength (bars) of foraging visits by the respective pollinators (a–i), with larger size indicating greater visitation. The adjoining rectangles, with white dot, indicate the flower-handling time of the respective pollinators on the morph. Only *Apis laboriosa* (blue bars) was the common forager among the two morphs. The relative strength of the links and bars (node), based on dependency-data from direct field observations (*see* **Table 2**), was generated by employing bipartite package (Dormann et al., 2008) in R ver. 4.3.2 (R Core Team, 2023). **(A,** a**)** butterfly (*Aglais caschmirensis aesis*); **(B,** b**)** bumblebee species; **(C,** c**)** hoverfly (*Scaeva pyrastri*); **(D,** d**)** dronefly (*Eristalis tenax*); **(E,** e**, F,** f**)** Himalayan giant honeybee (*Apis laboriosa*); **(G,** g**)** Chestnut-crowned laughingthrush (*Trochalopteron erythrocephalum*); **(H,** h**)** Streaked laughingthrush (*Trochalopteron lineatum*); **(I,** i**)** Himalayan bulbul (*Pycnonotus leucogenys*).

The group of floral visitors that interacted with the two morphs differed significantly (**Figure 1**; **Table 2**). In the red morph, all the floral visitors that visited flowers were represented by bird species namely Chestnut-crowned laughingthrush (*Trochalopteron erythrocephalum*, Leiothrichidae), Streaked laughingthrush (*Trochalopteron lineatum*, Leiothrichidae), and Himalayan bulbul (*Pycnonotus leucogenys*, Pycnonotidae) except for the Himalayan giant honeybee (*Apis laboriosa*, Apidae). Among these foragers, the birds were more effective in contacting the stigma at this elevation (**Table 2**). The Chestnut-crowned laughingthrush showed the highest average flower visits on a tree (7.70 ± 0.33, 5-12) followed by the Streaked laughingthrush (6.70 ± 0.33, 3-12). The Himalayan bulbul had the shortest flower-handling time (8.90 ± 0.49s, 5-14s) and the Himalayan giant honey bee had the longest flower-handling time (23.73 ± 1.99s, 10-48s). The foraging duration of the birds varied, with some foraging in the morning as well as evening and others foraging during the day. On the other hand, in the pink morph, insects were the major floral visitors namely *Apis laboriosa*, hoverfly (*Scaeva pyrastri*, Syrphidae), dronefly (*Eristalis tenax*, Syrphidae), butterfly (*Aglais caschmirensis aesis*, Nymphalidae) and a bumblebee species (Apidae). The Himalayan giant honeybee had the highest average number of flowers visits per tree (5.03 ± 0.26; 2-8) followed by dronefly (3.60 ± 0.19, 2-5). The Himalayan giant honeybee exhibited the longest flower-handling time (22.21 ± 1.74s, 4-40s) followed by the dronefly (12.63 ± 0.74, 6-22s) and then hoverfly (10.23 ± 0.43, 7-15s). Among these insects, *A. laboriosa* was most effective in foraging as pollinator (**Table 2**). The foraging duration of the pollinators varied, with the majority foraging during the morning hours (07:30-12:00h).

**Table 2:** Foraging behavior of different floral visitors on the two morphs of *R. arboreum*.

Species	Flowers visited per tree (n)	Flower-handling time (sec) (n)	Legitimacy* (n)	Trees visited per bout (n)	Foraging duration (h)	Peak foraging time (h)
Red Morph						
Chestnut-crowned laughingthrush (*Trochalopteron erythrocephalum*)	7.70 ± 0.3^a^ (30)	16.68 ± 0.7^b^ (30)	100 (30)	2.90 ± 0.2 (10)	07:30-10:00 16:00-18:00	08:00-09:30
Streaked laughingthrush (*Trochalopteron lineatum*)	6.70 ± 0.3^a^ (30)	16.05 ± 0.7^b^ (30)	100 (30)	2.60 ± 0.2 (10)	08:00-10:00 16:00-18:00	08:30-09:30
White-cheeked bulbul (*Pycnonotus leucogenys*)	6.67 ± 0.2^a^ (30)	8.90 ± 0.5^c^ (30)	100 (30)	2.40 ± 0.2 (10)	08:30-10:30	09:00-10:00
Himalayan giant honeybee (*Apis laboriosa*)	4.50 ± 0.19^b^ (30)	23.73 ± 2.0^a^ (30)	83.33 (30)	2.30 ± 0.1 (10)	10:00-13:00	10:30-12:00
Pink Morph						
Himalayan giant honeybee (*Apis laboriosa*)	5.03 ± 0.26^a^ (30)	22.21 ± 1.7^a^ (30)	86.67 (30)	2.40 ± 0.2 (10)	9:00-15:00	9:30-11:30
Hoverfly (*Scaeva pyrastri*)	3.00 ± 0.19^b^ (30)	10.23 ± 2.3^b^ (30)	70 (30)	2.00 ± 0.2 (10)	9:00-12:00	9:30-11:30
Dronefly (*Eristalis tenax*)	3.60 ± 0.19^b^ (30)	12.63 ± 0.7^b^ (30)	66.67 (30)	1.90 ± 0.2 (10)	9:00-12:00	9:30-11:30
Indian tortoiseshell Butterfly (*Aglais caschmirensis aesis)*	1.50 ± 0.5^b^ (2)	10.00 ± 5.0^ab^ (2)	50 (2)	1.00 ± 0.0 (2)	11:00-13:00	11:30-12:00
Bumblebee species	1.50 ± 0.5^b^ (2)	4.50 ± 0.5^b^ (2)	50 (2)	1.00 ± 0.0 (2)	9:00-10:00	9:30-10:00

*Legitimacy of interaction with flowers was estimated in terms of visits (%) accompanied by touch with the stigmatic surface. Different letters indicate significant differences by Tukey’s test and a pairwise comparison at a 5% level of significance

### Nectar analysis

The average standing-nectar volume in the red morphs was 179 ± 8 µL while in the pink it was 137 ± 5 µL; the difference was significant (**Supplementary Table 3**). The total sugar content was 27% and 30% in the red and pink morphs, respectively. Amino acid content was estimated to be 0.24 mg/mL in the red and 0.48 mg/mL in the pink morph (**Supplementary Table 5**). The nectar of the red morph was rich in total phenolics, while in the pink morph, it was noticed in traces. Alkaloids and proteins were not detected in either of the morphs.

### Outcrossing rate

The multi-locus (t_m_) outcrossing rate was 0.762 and 0.825 for the pink and red morphs, respectively, while single-locus (t_s_) was 0.750 and 0.824, respectively (**Table 3**). The value among the pink morphs lies between 0.2 and 0.8, and qualifies for a mixed-mating strategy (Goodwillie et al., 2005). The biparental inbreeding (t_m_- t_s_) was 0.012 among the pink morphs and it was 0.001 for the red morphs. The selfing rate (1-t_m_) was higher among the pink morphs (0.238) than that in the red morphs (0.175). The parental F estimate was more on the negative side (-0.200) in the pink morphs while it was 0.166 among the reds. The correlation of paternity (r_p_), as well as that of selfing among the loci (r_s_), was greater among the pink morphs (0.418; 0.804) than that observed among the reds (0.366; 0.699). Overall, the results suggest that both populations have low levels of inbreeding, though the selfing rate was higher among the pink morphs.

**Table 3:** Different estimates for mating system analysis among the two morphs of *R. arboreum*.

Morph	t_m_ [multi-locus t estimate]	t_s_ [Single-locus t estimate]	t_m_- t_s_ [Biparental inbreeding]	1-t_m_ [Selfing rate]	Parental F-estimate	r_p_ [Correlation of paternity]	r_s_ [Correlation of selfing among loci]
Pink	0.762 (0.080)	0.750 (0.098)	0.012 (0.042)	0.238	-0.200 (0.164)	0.418 (0.069)	0.804 (0.251)
Red	0.825 (0.028)	0.824 (0.052)	0.001 (0.045)	0.175	0.166 (0.169)	0.366 (0.104)	0.699 (0.248)

## Discussion

The present study demonstrates that the two distinct elevational floral morphs of *R. arboreum* exhibit variability in a range of reproductive attributes and preferences for pollinators that belong to two different functional groups of animals. Interestingly, the plant-pollinator interaction in the two morphs appears to elicit differences in the mating strategy.

### Reproductive attributes

#### Flowering phenology

Reproductive phenoevents comprising flowering, fruiting, and seed-dispersal can be strongly selected by temporal variations in the availability of interacting vectors (Augspurger, 1981). The phenoevents, especially the onset of flowering, are also known to be influenced by differences in temperature, and snowmelt patterns in the case of alpine plant communities (Arroyo et al., 2021). The usual trend is that the plants tend to bloom at the lower elevation first due to conducive conditions triggered by salubrious temperature and photoperiod (López-López et al., 2021). The two morphs in *R*. *arboreum* were phenologically separated due to similar reasons, especially in terms of the onset of flowering, with reds blooming earlier than the pinks. This distinction between the two morphs has never been highlighted earlier, though overlapping of phenophases such as blooming, fruiting, and seed dispersal in the species has been noted (Paul et al., 2018). It has been reported that the vegetative phenology (leaf initiation and leafing) in the species also follows a similar trend wherein events at higher elevations or tree-line are delayed by about two months (Singh and Negi, 2018). Besides the ambient temperature conditions, root-apex vernalization stimulus triggered by low soil-temperature has been linked to the onset of flowering among rhododendrons occupying higher elevations (Chandra et al., 2022). Interestingly, the duration of the flowering period among the two morphs was least affected by changes in elevation in our study. These findings are in line with earlier observations, especially for the red morphs, at other locations across Himalaya including Nepal (Ranjitkar et al., 2013), Kumaun, Central Himalaya (Singh et al., 2015), and Mizoram (Malsawmkima and Sahoo, 2021).

#### Floral biology

Structurally, the species did not show difference in the overall shape of the flower. However, the flower size in the two morphs differed significantly, especially in terms of the tube length. In red morphs the tube length was longer than that in the pink ones (**Supplementary Table 3**). Besides the floral size, there is pronounced herkogamy in the red morphs, as their style was significantly longer than the pink ones. Such adaptive floral attributes not only can cause strong selection on pollinators for a phenotypic matching but also enhance the pollination efficiency (Kenrick et al., 1987; Tandon et al., 2001; Thomson and Wilson, 2008). The pinks tend to invest less in pollen and ovule production than the reds, without compromising the pollen:ovule ratio per flower. The pinks also tend to compensate for this difference by investing more through greater flower production in an inflorescence. Such differences largely appear to contribute to the greater display required in pinks under alpine conditions, owing to a restricted pollinator activity as well as diversity (Kudo, 1991).

The flower structure in both the morphs is designed for enhanced outcrossing. Although the early onset of the stigma receptivity indicates protogyny, it was incomplete, as the male and female phases overlapped considerably over the lifespan of the flower. Occurrence of incomplete protogyny has been shown in *R. pulchrum* Sweet (Qiu et al., 2021), in contrast to *R. semibarbatum* Maxim., which exhibits protandry (Ono et al., 2008). The continuous receptivity of the stigma across the floral life-span could be due to the selective pressure to enhance the likelihood of receiving pollen, rather than aiding in pollen dispersal which in turn determines the longevity of flowers as seen in Flame Azalea (*R. calendulaceum* (Michx.) Torr.) (Blair and Wolfe, 2007). There is also the presence of marked herkogamy where the stigma is longer than even the longest stamen. The varying stamens’ lengths in *R. arboreum* flowers can attract a broader range of pollinators, each of which may have a different body size and foraging behavior. This diversifies the pool of potential pollinators and increases the chances of cross-pollination. Variability in the reproductive traits observed across different sites is likely a result of phenotypic adjustments to the diverse microhabitat conditions, rather than being primarily governed by genetically predetermined mechanisms, as demonstrated in prior studies (Pornon et al., 1997).

#### Nectar composition

Nectar serves as the primary floral reward among flowering plants (Baker and Baker, 1990), and our study emphasizes how differences in nectar concentration, composition, and quantity are reflected in the visitation rates of the pollinators at different elevations. Such attributes in floral suites are known to cause shift in pollinator assemblages, and shifting, particularly from bee to bird and vice-versa, has happened frequently and on independent occasions in the evolutionary history (van der Niet and Johnson, 2012; Rosas-Guerrero et al., 2014). It has also been argued that multi-trait convergence of flowers for a particular pollination syndrome result in better mutualistic interaction rather than altering of a single floral trait (Castellanos et al., 2004; Thomson and Wilson, 2008). Our study revealed that the pigmentation and morphometric differences in the two morphs are also accompanied by that in quantity and quality of the nectar reward. The red morphs produce copious amounts of nectar while the pink morphs have high amino acids along with sugars. These differences together might function well in accordance with the requirements of pollination guilds at the two elevations.

Previous studies on the nectar of *R. arboreum* (red morph) revealed lower sugar content. The presence of acetylandromedol (the main toxic principle with antibiotic properties) possibly compensates for lower sugar contents (Martini et al., 1990). These nectar attributes are in agreement with earlier observations that nectar-feeding passerine birds tend to forage flowers with lower nectar volume and higher sugar content, while those adapted for generalist bird pollinators typically have copious amounts of nectar volumes with low sugar content (Johnson and Nicolson, 2008).

### Pollination

In alpine ecosystems, varying snowmelt habitats along a cline can lead to significant differences in pollination success, fostering a diverse range of interactions between plants and pollinators (Kudo, 2022). The other species of *Rhododendron* are known to be pollinated by a range of pollinators encompassing birds, honey bees, bumblebees, butterflies, and flies (Stout, 2007; Kudo et al., 2011; Huang et al., 2017). Some of these species display geographic mosaic as the same plant species may be pollinated by a range of different pollinators at different altitudes. For example, *R. cyanocarpum* (Franch.) W.W.Sm. is pollinated by bumblebees in populations located at 3200 m and by birds above 3400 m (Ma et al., 2015; Huang et al., 2017).

In *R*. *arboreum*, the avian pollinators dominated at the lower elevations, and the role was replaced by insects at higher altitudes. This trend conforms with that of the other species of *Rhododendron* occupying the eastern Himalaya with no difference in the functional group of animals involved, although the species differed (Basnett et al., 2019). Also, sympatry among the two morphs was lacking and they were rather discrete at the two elevations in terms of pollinator assemblages (except *Apis laboriosa* Smith) as well. This suggests that the infraspecific variation in pollination guilds in the species is primarily driven by the elevational variability in the availability of floral foragers. The local adaptations of respective floral traits then refine the pollinator assemblages.

In general, plants engage insects as successful pollinators at higher elevations (Peeters and Totland, 1999). Among the insects, there is a general trend of increased prevalence of dipterans (flies) at higher elevations, while hymenopterans (bees) are predominating at the mid-elevations (Lefebvre et al., 2018). In our study, honeybees and flies (Syrphidae) serve as the primary pollinators at the higher elevation. In the western Himalayas, honeybees are reported as efficient pollinators where they are also the most abundant wild hymenopterans (Gautam et al., 2022). Here, bees generally prefer foraging on floral types associated with higher sugar concentration in nectar to ensure energetically advantageous foraging (Hill et al., 2001). Besides the bees, hoverflies have also been recognized as effective pollinators (as in *Rhododendron prattii* Franch.) at higher elevations (~3800 m) (Haiqing et al., 2021).

The red morphs with large showy flowers produce large amounts of relatively diluted nectar, which is rich in phenolics; these traits follow ornithophily (Faegri and van der Pijl, 1979). In Langtang National Park, Central Himalaya, Nepal, several species of birds have been recorded to visit *R. arboreum* mainly for nectar consumption (Ollerton et al., 2020). In our work, the Chestnut-crowned laughing thrush, Streaked laughingthrush, and Himalayan bulbul were the most frequent pollinators. Larger flowers at lower elevations, as indicated by the morphometry of the floral morphs, align with observations that they are more likely to adapt to bird pollination. While foraging, the birds’ nape/crown and bees’ bodies become fully enveloped within the corolla. In large-flowered *Rhododendron* species, effective pollen transfer depends on having a phenotypic match for larger animals for an enhanced legitimacy (Armbruster et al., 2011). Previous observations have shown that bees, due to their smaller size, often fail to encounter the stigma, causing a phenotypic mismatch (Huang et al., 2017). Also, bee visits in larger flowers typically result in significant pollen removal but limited pollen deposition on stigmas, leading to an inefficient pollination process (Wilson and Thomson, 1991). In contrast, larger animals such as birds and often some Lepidoptera (butterflies and large moths) are more likely to facilitate efficient pollen transfer due to their foraging behavior. In *R. arboreum*, the foraging behavior of the passerine birds involving perching, and leaning forward to access nectar from the base of the corolla, is conducive to effect pollination.

### Breeding system and outcrossing rate

Experimental pollinations clearly established that *R. arboreum* is a self-compatible species at the sites studied but invariably relies on pollinators for reproductive success. The tree species is a predominant outbreeder, as evidenced by a significantly reduced fruit-set from facilitated autogamy. The observed values of OCI and pollen:ovule ratio reiterate the outcrossing nature of the species. Prevalence of self-compatibility is also reported in other outbreeding species of *Rhododendron* like *R. aureum* Georgi (Kudo et al., 2011) and *R. cyanocarpum* (Ma et al., 2015) for ensuring reproductive assurance.

Difference in fruit-set between the open- and facilitated cross-pollinations gives an idea of pollen or pollinator limitation. In alpine system this limitation is more pronounced and may vary among species to different extents (Bingham and Orthner, 1998; Larson and Barrett, 2000; García-Camacho and Totland, 2009; Jiang and Xie, 2020). In our study, fruit-set from facilitated xenogamy was marginally higher but significant, than that from open-pollinations, among both the morphs, suggesting the limitation to some extent. However, an unusually high fruit-set through open-pollination could be attributed to release of pollen in compound units of tetrads that are further tangled by viscin threads, leading to mass deposition on the stigmatic surface. The lack of fruit-set resulting from spontaneous self-pollination can be attributed to the fact that *R. arboreum* flowers exhibit marked herkogamy, making self-pollination achievable only with the involvement of pollinators.

While self-pollination can be crucial in harsh environments with a limited window for random pollination, cross-pollination ensures increased genetic diversity within populations (Bliss, 1962). The outcrossing rate in *R. aureum* was about 80% regardless of season (Kudo et al., 2011). In *R. ferrugineum* L., a mixed-mating strategy was observed where abundant flower visitation rates (because of mate limitation) in smaller plant clusters led to low pollen limitation and increased rates of self-fertilization, whereas pollinator-limitation because of fewer flower visits in larger plant clusters resulted in heightened pollen limitation and greater outcrossing rates (Delmas et al., 2015). This study, including the present work, demonstrates that there can be substantial variation in the values of multi-locus outcrossing rates (t_m_) among different populations of the same species.

In *R*. *arboreum* a t_m_ of 0.82 among the red morphs points towards an outcrossing strategy while that of 0.76 among the pink morphs indicates affinity towards mixed-mating strategy (Goodwillie et al., 2005). These slight differences in the outcrossing rate might have resulted from the demographic and ecological variations between the two elevations, the main factor being the pollinators. Insect foraging behavior especially at the higher elevation may promote geitonogamous self-pollination, as the abundance of flowers in an inflorescence often leads to increased foraging within the genet, reducing the need for inter-genet visits (Stout, 2007). Conversely, at lower elevations, birds with their trapline foraging behavior may play a more significant role in effecting cross-pollination. In both these morphs, self-fertilization seems to be the primary contributor to inbreeding. This conclusion is drawn from the fact that the minimum variance means of the single-locus outcrossing estimate, t_s_, closely resembled the multi-locus estimate and exhibited a high correlation among loci, which was statistically not very different from 1 (Ritland, 2002).

Variations in pollinator guilds between the examined populations may offer insights into the disparities in outcrossing rates observed among the morphs of *R. arboreum*. The correlation of outcrossed population paternity (r_p_) reflects the likelihood that two siblings are full siblings resulting from outcrossing (Ritland, 2002). Both the studied populations show intermediate levels of correlation in paternity, indicating that in these populations, each tree has a limited number of potential parents or expected pollen donors, and outcrossing occurs in a non-random manner up to a certain extent or there is a presence of full siblings. The 1/r_p_ ratio, which provides an estimate of the likely number of effective pollen donors, was 2.4 among the pink morphs, suggesting that on average two donors participated in each cross, while it would be around three donors among the reds (2.7).

Pollen production among the *R. arboreum* trees is profuse and the male fitness enhances through the involvement of several pollinators by dispersing them to long distances. The substantial movement of pollen could also account for the limited occurrence of biparental inbreeding, as indicated by the marginal difference between multi-locus and single-locus outcrossing estimates. Based on the *F* estimates, the level of heterozygosity among the pink morphs (-0.200) is equal to or exceeds what would be expected from the Hardy-Weinberg equilibrium (Del Castillo and Trujillo, 2008). On the other hand, in the red morphs, the *F* estimate (0.166) signifies the presence of inbreeding in the maternal generation and affirms that *R. arboreum* is a species with a moderate to high degree of outcrossing (Sánchez-González et al., 2020).

## Conclusion

The two morphs of *R*. *arboreum* inhabit distinct elevational niches characterized by necessary conditions for inducing flowering. They exploit the available local fauna and establish mutualistic relationships to enhance reproductive success. The incidence of mixed-mating in the morph occupying higher elevations reflects beginning of a likely shift in reproductive strategy in a self-compatible yet predominantly outbreeding species. The distinction in functional floral traits among the two morphs and respective pollination guilds can possibly set the stage for divergence selection. However, demonstrating reproductive isolation among the morphs would require at-least proving the sterility of intermorph progeny.

## Data availability statement

The original contributions presented in the study are included in the article/**Supplementary Material**. Further inquiries can be directed to the corresponding author.

## Author contributions

SS: Data curation, Formal analysis, Investigation, Methodology, Software, Writing – original draft. AC: Data curation, Formal analysis, Validation, Writing – review & editing. RB: Data curation, Software, Validation, Visualization, Writing – review & editing. SB: Project administration, Resources, Writing – review & editing. SG: Formal analysis, Methodology, Resources, Validation, Writing – review & editing. RT: Conceptualization, Data curation, Formal analysis, Funding acquisition, Investigation, Methodology, Resources, Supervision, Validation, Visualization, Writing – original draft, Writing – review & editing.
