# Supplementary material for: Contradistinctive floral attributes, pollination guilds and their consequence on the outcrossing rate in two elevational morphs of Rhododendron arboreum Sm
Source: Front Plant Sci. 2024 Mar 28;15:1355680. doi: 10.3389/fpls.2024.1355680 (PMC11007036; doi:10.3389/fpls.2024.1355680)
Supplement: Supplementary file 1 [file Table_1.docx]

**Supplementary Table1**. Details of the different floral stages of *Rhododendron arboreum* recognized in the work.

| **Floral Stage** | **Peroxidase activity (bubbles/min)** | | **Key Floral Features** |
| --- | --- | --- | --- |
|  | **Red Morph (n=9)** | **Pink Morph**  **(n=9)** |  |
| R1 | 28.89±1.80 | 29.11±1.98 | 3 days before anthesis, small size bud, anthers not dehisced |
| R2 | 73.11±3.94 | 70.78±3.32 | 2 days before anthesis, medium size bud, undehisced anthers |
| R3* | 117.78±5.60 | 121.89±5.44 | 1 day before anthesis, large size bud, undehisced anthers |
| R4* | 161.89±7.63 | 164.44±5.55 | Anthetic stage, petals begin to unfurl at the tip, anther dehiscence also begins |
| R5 | 115.33±5.01 | 120.33±2.07 | 1-2 days after anthesis, petals fully open, |
| R6 | 6.11±1.45 | 5.56±1.39 | 3-4 days after anthesis, petals withered |

**Stages used for pollination treatments*

**Supplementary Table 2.** Details of the 7 SSR primer pairs used in the study (Sharma et al., 2020).

| **S. No.** | **Marker name** | **Repeat motif** | **Forward Primer sequence (5’-3’)** | **Reverse primer sequence (5’-3’)** | **Ta (°C)** | **Size Range** |
| --- | --- | --- | --- | --- | --- | --- |
| 1 | RHC_MS8 | (CCA)6 | TACTTTTCCCAACACTCCTCT | TAGAAATGTCACGTACGCTCT | 55 | 200–400 |
| 2 | RHM_MS2 | (CA)7 | AGATATTTTGGTTTCCCCTCT | AGCAGCATTAAATAGGCATAA | 51 | 150–300 |
| 3 | RHC_MS5 | (CT)9 | TGCACATAGAACACAAAATCA | CTATGCCACCAACTTTGTC | 52 | 140–700 |
| 4 | RHC_MS11 | (GTCT)4 | CTTGAGTGAGAGAGAGCATTG | TATGACACATGACAGAGAGCA | 55 | 150–500 |
| 5 | RHC_MS12 | (ATACAA)3 | AGCAGACTATATGCAAAAGCA | TATCGCATGTTGGTTTAATTC | 52 | 150–600 |
| 6 | RHM_MS3 | (TG)7 | ATCCAACCTTTGTAGGATCAC | CAAACGATAGAAGACGGTTTA | 53 | 150–500 |
| 7 | RHM_MS5 | (TC)8 | GATTTTCTCCAATCAACATCA | CATCTAACAGATCGAGCAGAC | 52 | 100–350 |

**Supplementary Table 3**. Different floral parameters compared between the two morphs (n=20, each morph). P value is based on 0.05 probability level.

| **Floral Attributes** | **Red Morph** | **Pink Morph** | **P value (T-test)** |
| --- | --- | --- | --- |
| No. of flowers in an inflorescence | 14.6 ± 0.4 | 17.4 ± 0.6 | 0.001 |
| Corolla width (cm) | 5.1 ± 0.09 | 4.9 ± 0.07 | 0.040 |
| Length of Corolla tube (cm) | 4.4 ± 0.05 | 3.6 ± 0.10 | 0.000 |
| Pistil Length (cm) | 5.0 ± 0.05 | 3.9 ± 0.04 | 0.000 |
| Longest Stamen Length (cm) | 3.7 ± 0.05 | 3.3 ± 0.05 | 0.000 |
| Shortest Stamen length (cm) | 2.5 ± 0.04 | 2.0 ± 0.05 | 0.000 |
| Pollen fertility (%) | 99.3 ± 0.2 | 99.4 ± 0.2 | 0.744 |
| Pollen viability (%) | 83.3 ± 1.1 | 83.9 ± 1.2 | 0.711 |
| Pollen production in a flower | 858900 ± 11976 | 655750 ± 8459 | 0.000 |
| Ovule production in a flower | 3670 ± 39 | 2866 ± 55 | 0.000 |
| Pollen: Ovule ratio | 235 ± 5 | 231 ± 6 | 0.572 |
| Number of seeds in a capsule | 1627 ± 81 | 1092 ± 40 | 0.000 |
| Seed: Ovule ratio (% seed-set) | 0.44 ± 0.02 (44) | 0.38 ± 0.02 (38) | 0.044 |
| Standing Nectar Volume (µL) | 179 ± 8 | 137 ± 5 | 0.000 |

**Supplementary Table 4**. Output of indices of breeding system in *R*. *arboreum.*

| **Indices** | **Red morph** | **Pink Morph** |
| --- | --- | --- |
| Index of self-incompatibility (ISI) | 0.63 | 0.74 |
| Pollen Limitation | 0.13 | 0.09 |
| Reproductive Efficacy | 0.87 | 0.91 |
| Inbreeding Depression (fruit-set) | 0.37 | 0.26 |

**Supplementary Table** **5**. Details of nectar composition among the two floral morphs of *Rhododendron arboreum*.

| **Compounds** | **Red Morph (n=10)** | **Pink Morph (n=10)** |
| --- | --- | --- |
| Total Sugar | 27% | 30% |
| Amino Acids | 0.24375 mg/mL | 0.4875 mg/mL |
| Phenolics | ++ | + |
| Alkaloids | - | - |
| Proteins | - | - |
